# Association of the presence of allergic disease with subsequent risk of liver cancer in a nationwide retrospective cohort among Koreans

**DOI:** 10.1038/s41598-022-14147-4

**Published:** 2022-06-14

**Authors:** Ji Ah Kim, Sun Jae Park, Seulggie Choi, Jooyoung Chang, Seogsong Jeong, Joseph C.Ahn, Gyeongsil Lee, Joung Sik Son, Sang Min Park

**Affiliations:** 1 Department of Public Health, Graduate School of Public Health, Seoul National University, Seoul, South Korea; 2 Department of Biomedical Sciences, Seoul National University Graduate School, Seoul National University College of Medicine, 101 Daehak-ro, Jongno-gu, Seoul, South Korea; 3 Department of Biomedical Informatics, CHA University School of Medicine, CHA University, Seongnam, 13488 Korea; 4 Division of Gastroenterology and Hepatology, Mayo Clinic, Rochester, MN USA; 5 Department of Family Medicine, Seoul National University Hospital, Seoul, South Korea; 6 Department of Internal Medicine, Hallym University Sacred Heart Hospital, Anyang, South Korea

**Keywords:** Cancer, Immunology, Health care, Oncology

## Abstract

A number of studies have proposed an inverse association between allergic diseases and risk of cancer, but only a few studies have specifically investigated the risk of primary liver cancer, including hepatocellular carcinoma (HCC) and intrahepatic cholangiocarcinoma (ICC). The aim of this study was to evaluate the association of allergic diseases with risk of primary liver cancer. We conducted a retrospective cohort study of the Korean National Health Insurance Service database consisted of 405,512 Korean adults ages 40 and above who underwent health screening before January 1st, 2005. All participants were followed up until the date of liver cancer, death, or December 31st, 2013, whichever happened earliest. Those who died before the index date or had pre-diagnosed cancer were excluded from the analyses. Cox proportional hazards regression was used to determine the adjusted hazard ratios (aHRs) and 95% confidence intervals (CIs) for risk of primary liver cancer according to the presence of allergic diseases, including atopic dermatitis, asthma, and allergic rhinitis. The aHR (95% CI) for overall liver cancer among allergic patients was 0.77 (0.68–0.87) compared to those without allergic disease. Allergic patients had significantly reduced risk of HCC (aHR, 0.72; 95% CI 0.62–0.85) but not ICC (aHR, 0.95; 95% CI 0.73–1.22). The presence of allergies was associated with significantly lower risk of liver cancer among patients whose systolic blood pressure is lower than 140 mmHg (aHR, 0.64; 95% CI 0.62–0.78 for overall liver cancer; aHR, 0.64; 95% CI 0.52–0.78 for HCC) but this effect was not observed among patients whose systolic blood pressure is higher than 140 mmHg (aHR, 0.91; 95% CI 0.71–1.18 for overall liver cancer; aHR, 0.91; 95% CI 0.71–1.18 for HCC) The aHR (95% CI) for overall liver cancer of allergic patients with and without chronic hepatitis virus infection were 0.60 (95% CI 0.44–0.81) and 0.77 (95% CI 0.64–0.93), respectively. In addition, allergic patients without cirrhosis showed significantly lower risk of overall liver cancer (aHR, 0.73; 95% CI 0.63–0.83). Patients with allergic diseases have significantly lower risk of primary liver cancer compared to those without allergic diseases, which supports the rationale for immunotherapy as an effective treatment for liver cancer.

## Introduction

Liver cancer is one of the leading causes of cancer incidence and mortality globally. In South Korea, liver cancer is the sixth common cancer and the second largest cause of cancer mortality^1^. Liver cancer can be classified into primary and metastatic liver cancer^2^. Types of primary liver cancer include hepatocellular carcinoma (HCC), intrahepatic cholangiocarcinoma (ICC), and other unusual tumors. HCC accounts for the majority of primary liver cancer and ICC accounted for approximately 10% of liver cancers in South Korea^3^. HCC usually develops in the background of chronic viral hepatitis or liver cirrhosis from various causes. While HCC is potentially curative via resection, ablation, or liver transplantation if diagnosed at an early stage, most HCC patients are often diagnosed at advanced stages that lead to poor prognosis^4^. Despite the promising recent developments in the role of immunotherapy for HCC, the treatments for advanced HCC remain largely palliative^5^. Therefore, it is crucial to identify factors that modify the risk of HCC as they may provide insight into novel preventative or therapeutic options.

Atopic dermatitis, asthma, and allergic rhinitis are the most prevalent allergic diseases worldwide, and their physiology is defined by IgE-mediated immunological hyperresponsiveness. IgE mediates the type I hypersensitivity reactions engaged in the pathogenesis of allergic diseases and is also involved in immune responses against cancer^6,7^. Several studies have suggested an inverse association between allergic diseases and cancer development^8^. However, there are only a few studies that investigated the association between allergic diseases and risk of liver cancer, and these studies were limited by the scope allergic diseases investigated, short follow-up, small sample size, and no consideration of risk factors, such as hepatitis B virus (HBV) and hepatitis C virus (HCV) infection and cirrhosis^9–11^. Therefore, the aim of our study was to investigate the association between allergic diseases and the risk of liver cancer using a large population-based cohort from a national database with long term follow-up.

## Methods

### Data source and study design

In this retrospective cohort study, data was derived from the Korean National Health Insurance Service (NHIS) database, which covers information on almost all forms of healthcare services. In South Korea, the NHIS provides compulsory health insurance for nearly all citizens, accounting for 98% of the entire population^12^. Starting at age of 40, all citizens are eligible to undergo a biennial national health screening executed by the NHIS.

At the health screening, participants’ basic health information, including weight, height, blood pressure, fasting serum glucose, and total cholesterol level are recorded. Participants also fill out self-reported questionnaires about lifestyle behaviors, including smoking status, alcohol consumption, and physical activities. The NHIS collects and stores insured personal health information, such as sociodemographic features, inpatient and outpatient hospital visits, prescription records of pharmaceutical drugs, and all other laboratory measurements obtained from the national health screening examinations^13^. The NHIS database has been widely used for research purposes, especially in epidemiological studies and its validity is described elsewhere^12^.

This study was conducted upon the approval of the Institutional Review Board of Seoul National University Hospital (IRB number: E-1806-076-951). The requirement for informed consent was waived since the NHIS database is anonymized based on strict confidentiality guidelines before distribution. This study adhered to the principles of the Declaration of Helsinki and all methods were performed in accordance with relevant guidelines and regulations.

### Study population

**Figure 1:**
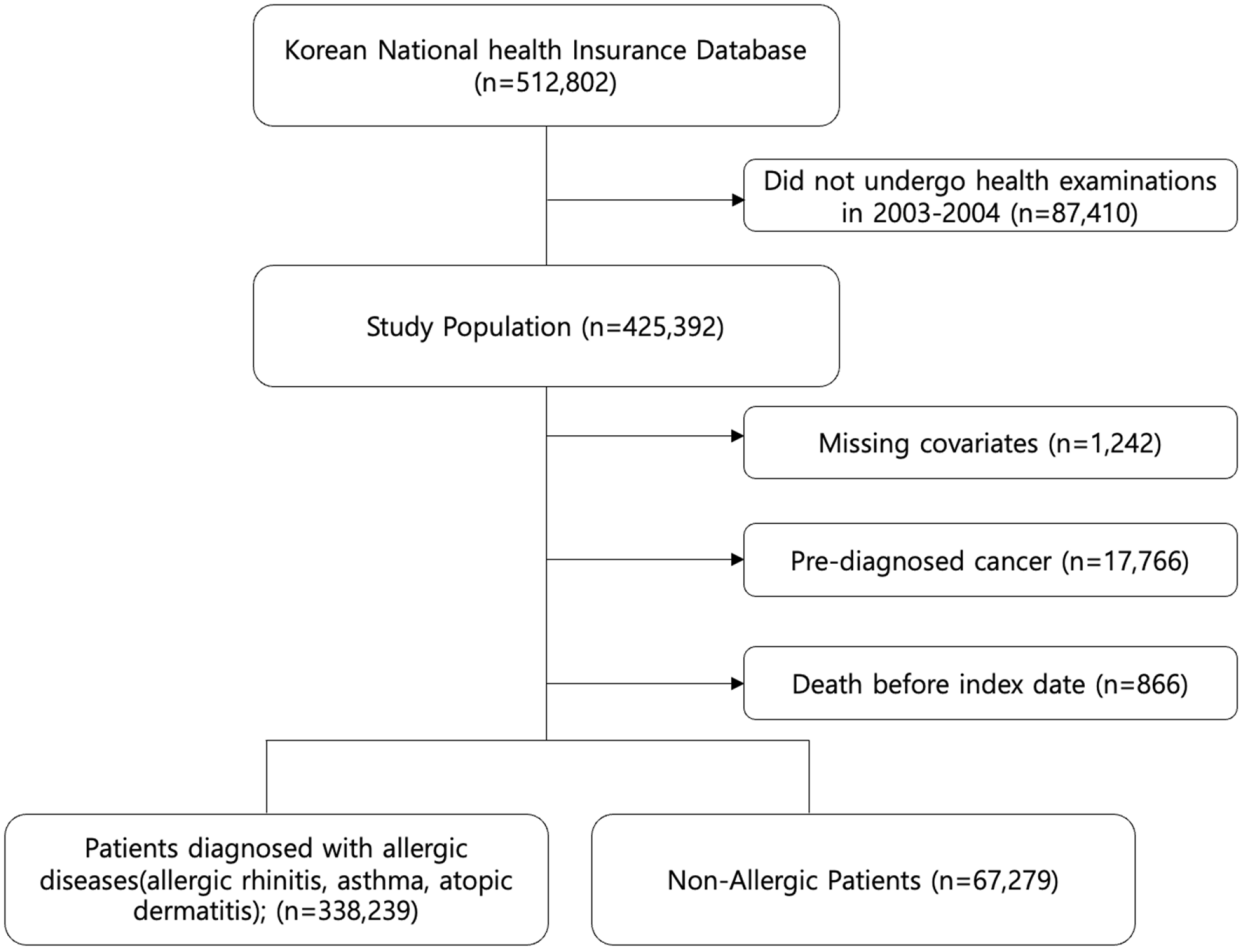
Flow diagram of the study population.

In this study, we identified all patients ages 40 and above who underwent the national health screening before the index date of January 1st, 2005. Those who did not undergo health screening examinations, missing values on covariates, history of cancer, death before the index date were excluded. The final study population included 405,512 participants, and all participants were followed up from January 1st, 2005 for liver cancer or death until December 31st, 2013 (Fig. 1). Death occurred for 28,865 participants before the date of December 31st, 2013 among participants without occurrence of primary liver cancer within follow-up period, and all other participants were followed up until December 31st, 2013.

### Key variables

#### Definition of liver cancer events

Based upon diagnosis and death codes, we defined liver cancer events as having outpatient medical records under related diagnostic codes for more than 3 times in a year or being hospitalized for 1 or more days or death under the corresponding ICD-10 codes. In this study, the international classification of diseases 10th revision (ICD-10) codes of C22, C220, and C221 were used for overall liver cancer, HCC, and ICC, respectively, as described in previous studies^14,15^.

#### Definition of the diagnosed history of allergic diseases

The allergic diseases (atopic dermatitis, asthma, and allergic rhinitis) were defined as having outpatient visits with relevant diagnostic codes for more than two times or with hospitalization for 1 or more days. Since allergic diseases mostly develop during childhood, we did not wash out previously confirmed cases but measured the prevalence of allergic diseases from 2002 to 2004. The ICD-10 codes were used to define atopic dermatitis (L20), asthma (J45), and allergic rhinitis (J30.1, J30.2, J30.3, and J30.4).

#### Ascertainment of covariates

Following potential confounding factors are included for the adjusted analyses: HBV/HCV infection (categorical; yes or no), liver cirrhosis (categorical; yes or no), age (continuous; years), sex (categorical; men and women), body mass index (continuous; kg/m2) , household income (categorical; 1st, 2nd 3rd, and 4th quartiles), Charlson comorbidity index (continuous), smoking status (categorical; never, former, and current smokers), alcohol consumption (categorical; 0, 1–2, 3–4, and ≥ 5 times per week), physical activity (categorical; 0, 1–2, 3–4, 5–6, and 7 times per week), systolic blood pressure (continuous; mmHg), fasting glucose serum (continuous; mg/dL), total cholesterol (continuous; mg/dL) and the total number of outpatient visits from 2002 to 2004 (continuous; times). For sensitivity analysis, the total number of outpatient visits (continuous; times) and the duration of hospitalization (continuous; days) only for allergic diseases were included. Body mass index was calculated by dividing the weight in kilograms by the square of height in meters. Household income was derived from the insurance premium and the calculation of Charlson comorbidity index was adapted from a previous study^16^.

Since the infection of either HBV/HCV and liver cirrhosis are major risk factors for liver cancer, we additionally considered those factors as covariates. Operational definitions of the HBV/HCV infection and liver cirrhosis were having outpatient department visits for more than 3 times or being hospitalized for 1 or more days. The ICD-10 codes of B180 and B181 were used for HBV, B182 was used for HCV, and I859, I982, K703, K717, and K746 were used for liver cirrhosis, respectively^17^.

#### Statistical analysis

Participants were evaluated for adjusted hazard ratios (aHRs) and 95% confidence intervals (CIs) for liver cancer risk using multivariate Cox proportional hazards regression. Five types of analyses were carried out. First, aHRs of HCC and ICC were separately calculated. Second, stratified analyses according to several potential covariates were conducted. Third, patients were classified based on the type of allergic diseases. Fourth, the association of the complexity of allergic diseases with liver cancer was investigated. Lastly, sensitivity analyses were performed by adjustments for the severity of allergic diseases.

Statistical significance was defined as a 2-sided P value of < 0.05. During study, data collection and statistical analyses were performed by using SAS 9.4 (SAS Institute, Cary, NC, USA) and STATA 13.0 (StataCrop LP, College Station, TX, USA). Kaplan–Meier curve was generated using R version 4.1.1 (www.r-project.org).

## Results

Descriptive characteristics of the study population are depicted in Table 1. Among the overall cohort of 405,512 participants, there were 338,239 without history of allergies and 67,279 with documented allergic diseases. 3059 (0.75%), 21,487 (5.30%), and 49,716 (12.26%) participants had atopic dermatitis, asthma, and allergic rhinitis, respectively. Some participants had multiple allergies and were included in more than one allergic disease categories. Compared with participants who have not been diagnosed with allergic diseases, patients with allergic diseases tended to be older, female, smoke less, consume less alcohol, have more comorbidities and higher prevalence of HBV/HCV infection.

**Table 1:** Descriptive characteristics of the study population.

	Total	Non-allergic patients	Allergic patients	Atopic dermatitis	Asthma	Allergic rhinitis
Participants, N (%)	405,518 (100)	338,239 (83.41)	67,279( 16.59)	3059 (0.75)	21,487 (5.30)	49,716 (12.26)
**Total cohort**						
**Age, years, N (%)**						
40–49	163,337 (40.28)	137,791 (40.74)	25,546 (37.97)	1093 (35.73)	5186 (24.14)	21,390 (43.02)
50–59	126,400 (31.17)	106,714 (31.55)	19,686 (29.26)	993 (32.46)	5873 (27.33)	14,900 (29.97)
60–69	84,407 (20.81)	68,904 (20.37)	15,503 (23.04)	673 (22.00)	6776 (31.54)	10,013 (20.14)
≥ 70	31,374 (7.74)	24,830 (7.34)	6544 (9.73)	300 (9.81)	3652 (17.00)	3413 (6.86)
**Sex, N (%)**						
Men	223,494 (55.11)	191,288 (56.55)	32,206 (47.87)	1649 (53.91)	9313 (43.34)	24,226 (48.73)
Women	182,024 (44.89)	146,951 (43.45)	35,073 (52.13)	1410 (46.09)	12,174 (56.66)	25,490 (51.27)
**Household income, quartile, N (%)**						
1st (highest)	141,931 (35.00)	117,520 (34.74)	24,411 (36.28)	1147 (37.50)	6712 (31.24)	19,030 (38.28)
2nd	116,551 (28.74)	97,371 (28.79)	19,180 (28.51)	844 (27.59)	6180 (28.76)	14,110 (28.38)
3rd	84,547 (20.85)	71,220 (21.06)	13,327 (19.81)	622 (20.33)	4658 (21.68)	9496 (19.10)
4th (lowest)	62,489 (15.41)	52,128 (15.41)	10,361 (15.40)	446 (14.58)	3937 (18.32)	7,080 (14.24)
**Charlson comorbidity index, N (%)**						
0 or 1	282,464 (69.66)	246,550 (72.89)	35,914 (53.38)	1822 (59.56)	7319 (34.06)	29,219 (58.77)
> 1	123,054 (30.34)	91,689 (27.11)	31,365 (46.62)	1237 (40.44)	14,168 (65.94)	20,497 (41.23)
**History of HBV/HCV infection, N (%)**^**b**^						
No	397,868 (98.11)	332,055 (98.17)	65,813 (97.82)	2990 (97.74)	21,036 (97.90)	48,598 (97.75)
Yes	7650 (1.89)	6184 (1.83)	1466 (2.18)	69 (2.26)	451 (2.10)	1,118 (2.25)
**History of liver cirrhosis, N (%)**						
No	403,424 (99.48)	336,484 (99.48)	66,940 (99.50)	3041 (99.41)	21,362 (99.42)	49,481 (99.53)
Yes	2094 (0.52)	1755 (0.52)	339 (0.50)	18 (0.59)	125 (0.58)	235 (0.47)
**Health screening cohort**						
Body mass index, kg/m^2^, mean (SD)	23.98	23.97	24.03	23.96	24.19	23.99
Systolic blood pressure, mmHg, mean (SD)	127.19	127.37	126.29	127.40	128.50	125.36
Total cholesterol, mg/dL, mean (SD)	199.68	199.52	200.47	202.00	201.93	200.03
Fasting serum glucose, mg/dL, mean (SD)	98.16	98.42	96.82	97.40	98.46	96.10
**Smoking status, N (%)**						
Never smoker	261,512 (65.47)	217,567 (64.32)	47,945 (71.26)	2,038 (66.62)	15,556 (72.40)	35,564 (71.53)
Former smoker	35,465 (8.75)	29,684 (8.78)	5781 (8.59)	280 (9.15)	1611 (7.50)	4,426 (8.90)
Current smoker	89,888 (22.17)	78,781 (23.29)	11,107 (16.51)	603 (19.71)	3559 (16.56)	7,909 (15.91)
Missing^a^	14,653 (3.61)	12,207 (3.61)	2446 (3.64)	138 (4.51)	761 (3.54)	1,817 (3.65)
**Physical activity, times per week, N (%)**						
0	213,965 (52.76)	178,898 (52.89)	35,067 (52.12)	1,623 (53.06)	12,433 (57.86)	24,767 (49.82)
1–2	99,126 (24.44)	83,320 (24.63)	15,806 (23.49)	687 (22.46)	4217 (19.63)	12,410 (24.96)
3–4	42,679 (10.52)	35,180 (10.40)	7499 (11.15)	344 (11.25)	1894 (8.81)	5,986 (12.04)
5–6	11,511 (2.84)	9411 (2.78)	2100 (3.12)	79 (2.58)	593 (2.76)	1,664 (3.35)
7	29,054 (7.16)	23,785 (7.03)	5269 (7.83)	247 (8.07)	1808 (8.41)	3,810 (7.66)
Missing^a^	9183 (2.26)	7645 (2.26)	1538 (2.29)	79 (2.58)	542 (2.52)	1,079 (2.17)
**Alcohol consumption frequency, N (%)**						
0	226,087 (55.75)	184,893 (54.66)	41,194 (61.23)	1793 (58.61)	14,287 (66.49)	29,726 (59.79)
< 1	60,796 (14.99)	50,904 (15.05)	9892 (14.70)	468 (15.30)	2617 (12.18)	7,747 (15.58)
1–2	67,114 (16.55)	57,617 (17.03)	9497 (14.12)	461 (15.07)	2430 (11.31)	7,440 (14.97)
3–4	27,826 (6.86)	24,377 (7.21)	3449 (5.13)	183 (5.98)	954 (4.44)	2,579 (5.19)
≥ 5	17,150 (4.23)	15,004 (4.44)	2146 (3.19)	109 (3.56)	817 (3.80)	1,438 (2.89)
Missing^a^	6545 (1.61)	5444 (1.61)	1101 (1.64)	45 (1.47)	382 (1.78)	786 (1.58)

^a^Participants with lifestyle related variables missing are marked.

^b^Having a history of hepatitis B/C virus infection was defined as having a disease code of either hepatitis B or hepatitis C described in the medical record of the participant.

The association of the history of allergic diseases with risk of liver cancer incidence were presented in Table 2 and Supplementary Table S1. During the follow-up period from January 1st, 2005 to December 31st, 2013, 2040 of non-allergic patients during a 2,890,854 person-year follow up and 310 of allergic patients during a 571,420 person-year follow up experienced liver cancer. Allergic patients had significantly lower risk of overall liver cancer compared to non-allergic patients (aHR, 0.77; 95% CI 0.68–0.87). Among the two main categories of primary liver cancer, the risk of HCC was significantly reduced in patients with allergic diseases (aHR, 0.72; 95% CI 0.62–0.85) but the risk of ICC had no association with allergic diseases (aHR, 0.95; 95% CI 0.73–1.22). Also, we performed sensitivity analysis and provided two other models by further adjusting the number of outpatient visits for allergic diseases and the duration of hospitalization for allergic diseases, respectively (Supplementary Table S1). Even we further considered the severity of allergic diseases, the inverse association of allergic diseases with either overall liver cancer(aHR, 0.73; 95% CI 0.62–0.86 in Model 1, aHR, 0.78; 95% CI 0.69–0.88 in Model 2) or HCC (aHR, 0.68; 95% CI 0.56–0.83 in Model 1, aHR, 0.73; 95% CI 0.63–0.86 in Model 2). The aHRs for ICC in both models were 0.87 (95% CI 0.63–1.19) and 0.95 (95% CI 0.74–1.23) without statistical significances.

**Table 2:** Association of the history of allergic diseases with risk of liver cancer incidence.

	Non-allergic patients	Allergic patients
**Overall**		
Events, N (%)	2,045 (0.60)	310 (0.46)
Person-years	2,890,854	571,420
aHR (95% CI)	1.00 (Reference)	0.77 (0.68–0.87)
**HCC**		
Events, N (%)	1,428 (0.42)	196 (0.29)
aHR (95% CI)	1.00 (Reference)	0.72 (0.62–0.85)
**ICC**		
Events, N (%)	402 (0.12)	78 (0.12)
aHR (95% CI)	1.00 (Reference)	0.95 (0.73–1.22)

Hazard ratio calculated by Cox proportional hazards regression analysis after adjustments for age, sex, body mass index, Charlson comorbidity index, household income, smoking, alcohol consumption, physical activity, systolic blood pressure, fasting serum glucose, total cholesterol, history of hepatitis B virus/hepatitis C virus infection, liver cirrhosis, and the number of outpatient visits.

*N* number of people, *aHR* adjusted hazard ratio, *CI* confidential interval, *HCC* Hepatocellular carcinoma, *ICC* intrahepatic cholangiocarcinoma.

**Figure 2:**
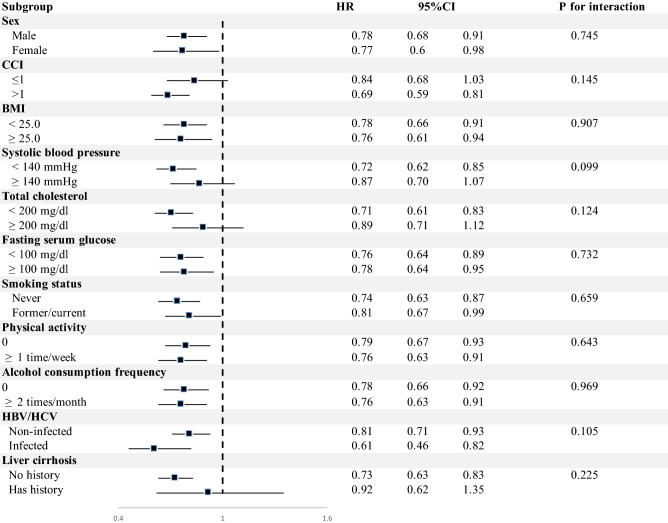
Stratified analysis on the association of the history of allergic diseases with the risk of liver cancer. Hazard ratio calculated by Cox proportional hazards regression analysis after adjustments for age, sex, body mass index, Charlson comorbidity index, household income, smoking, alcohol consumption, physical activity, systolic blood pressure, fasting serum glucose, total cholesterol, history of hepatitis B virus/hepatitis C virus infection and liver cirrhosis and the number of outpatient visits.

According to all covariates included in this study, stratified analyses were performed and plotted as shown in Fig. 2, Supplementary Fig. S1, and Supplementary Fig. S2. In all sub-group analyses, allergic patients show reduced hazard ratios for overall liver cancer and HCC. Mostly there was no significant interactions between allergic state and the risk of liver cancer across prespecified variables. In health screening cohort, body mass index, fasting glucose serum and alcohol consumption frequency which are risk factors fatty liver diseases showed decreased hazard ratios regardless of the history of allergic diseases^18,19^. Presence of allergies was associated with significantly lower risk of liver cancer among patients whose systolic blood pressure is lower than 140 mmHg (aHR, 0.64; 95% CI 0.62–0.78 for overall liver cancer; aHR, 0.64; 95% CI 0.52–0.78 for HCC) but this effect was not observed among patients whose systolic blood pressure is higher than 140 mmHg (aHR, 0.91; 95% CI 0.71–1.18 for overall liver cancer; aHR, 0.91; 95% CI 0.71–1.18 for HCC) The aHR (95% CI) for overall liver cancer of allergic patients infected and not infected with HBV/HCV virus were 0.60 (95% CI 0.44–0.81) and 0.77 (95% CI 0.64–0.93), respectively. Allergic patients without cirrhosis showed significantly lower risk of overall liver cancer (aHR, 0.73; 95% CI 0.63–0.83), compared to patients with cirrhosis (aHR, 0.92; 95% CI 0.62–1.35). Participants were further classified and analyzed depending on the types of allergic diseases (Table 3 and Supplementary Table S2). The aHR (95% CI) for atopic dermatitis, asthma, and allergic rhinitis were 0.92 (0.55–1.52), 0.80 (0.66–0.97), and 0.76(0.65–0.88) for overall liver cancer, and 0.80 (0.42–1.55), 0.71 (0.55–0.91), and 0.73 (0.60–0.87) for HCC, respectively.

**Table 3:** Association of allergic diseases with risk of liver cancer.

	Non-atopic dermatitis	Atopic dermatitis	Non-asthma	Asthma	Non-allergic rhinitis	Allergic rhinitis
**Overall**						
Events, N (%)	2,340 (0.58)	15 (0.49)	2,233 (0.58)	122 (0.57)	2,152 (0.60)	203 (0.41)
Person-years	3,436,318	25,956	3,284,814	177,459	3,035,449	426,824
aHR (95% CI)	1.00 (Reference)	0.92 (0.55–1.52)	1.00 (Reference)	0.80 (0.66–0.97)	1.00 (Reference)	0.76 (0.65–0.88)

Hazard ratio calculated by Cox proportional hazards regression analysis after adjustments for age, sex, body mass index, Charlson comorbidity index, household income, smoking, alcohol consumption, physical activity, systolic blood pressure, fasting serum glucose, total cholesterol, history of hepatitis B virus/hepatitis C virus infection and liver cirrhosis and the number of outpatient visits.

Finally, we investigated differences in liver cancer risk among patients with single allergic disease and those with multiple allergic diseases (Table 4 and Supplementary Table S3). In Table 4, compared to patients with no history of allergies, aHRs (95% CI) of atopic dermatitis only, asthma only, allergic rhinitis only, and two or more allergies were 0.98 (0.57–1.70), 0.80 (0.65–1.00), 0.76 (0.66–0.89), and 0.66 (0.45–0.97), respectively. Supplementary Table S3 also shows protective association of allergic diseases with risk of HCC in each patient group, but not for ICC. The aHRs (95% CIs) for HCC were 0.86 (0.43–1.73), 0.74 (0.55–0.98), 0.74(0.61–0.90), and 0.52(0.31–0.89), respectively. Patients with multiple allergic diseases had the lowest risk of liver cancer contrast to those with single allergic disease.

**Table 4:** Association of allergic disease incidence with risk of liver cancer incidence.

	No allergic history	Atopic dermatitis only	Asthma only	Allergic rhinitis only	Two or more allergies^a^
**Overall**					
Events, N (%)	2,045 (0.60)	13 (0.57)	94 (0.62)	174 (0.40)	29 (0.42)
Person-years	3,042,993	20,359	136,262	386,726	61,932
aHR (95% CI)	1.00 (Reference)	0.96 (0.56–1.66)	0.79 (0.63–0.98)	0.78 (0.66–0.91)	0.65 (0.44–0.96)

Hazard ratio calculated by Cox proportional hazards regression analysis after adjustments for age, sex, body mass index, Charlson comorbidity index, household income, smoking, alcohol consumption, physical activity, systolic blood pressure, fasting serum glucose, total cholesterol, history of hepatitis B virus/hepatitis C virus infection and liver cirrhosis and the number of outpatient visits.

^a^Having the history of allergic diseases of either any two kinds of allergies among allergic rhinitis, atopic dermatitis, asthma or all of three allergies.

## Discussion

Our large population-based longitudinal retrospective cohort study has demonstrated the protective effects of allergic diseases on risk of liver cancer, especially HCC. This phenomenon was observed across different types of allergic diseases, and especially more pronounced among those with multiple allergic diseases who presumably have more severe allergies. To our knowledge, this is the first study that clarified the association of allergic disease with risk of HCC by adjusting not only for strong risk factors, such as HBV/HCV infection and liver cirrhosis, but also body mass index, fasting glucose serum, and alcohol consumption frequency, which were previously found to be associated with alcoholic and nonalcoholic fatty liver diseases. The results remained consistent even after consideration of variables regarding medical resource utilization and the severity of allergic diseases by adjusting the number of outpatient visits and admission for allergic diseases.

The protective effect of allergic diseases was limited to patients without cirrhosis and presence of allergic diseases was not associated with reduction of HCC risk among patients with cirrhosis. This is likely due to the irreversible damage and fibrosis that have taken place in a cirrhotic liver, which predisposes to development of HCC^20^. Moreover, cirrhosis affects innate immunity by impairing the synthesis and function of related proteins^21^. This may attenuate the allergy-driven immune responses within the liver and remove the protective effects of allergic diseases. Also, the inverse association of allergic diseases with the risk of HCC was more attenuated among patients with higher blood pressure. This might because that patients with lower blood pressure are likely to have fewer confounding factors that can influence liver tumorigenesis. Possibly, there would be a potential underlying mechanism between high blood pressure and allergic disease on liver cancer development. Future studies are needed to clarify this relationship.

Many epidemiologic studies have shown an inverse relationship between cancer incidence and presence of allergic diseases in various types of cancers. The strong and negative association of allergic diseases with cancer risk particularly for pancreatic cancer, brain cancers, hematological cancers and gastrointestinal cancers was well addressed in several studies^22^. Some studies showed more highlighted significantly reduced risk^23–28^. Regarding liver cancer, the following allergic diseases showed inverse relationships with liver cancer in previous studies: history of drug allergies^9^, allergic rhinitis and asthma^10^, and hay-fever or allergic rhinitis^11^. All in all, it is well-acknowledged that there would be promising protective effects of allergic diseases on cancer development.

There are several underlying mechanisms that explain why allergic patients might be protected against cancer development. Two of the leading hypotheses include immunosurveillance and prophylaxis^29^. The “immunosurveillance hypothesis” explains that since allergy is a consequence of improved and hyper-responsive immune system, which enables easy recognition and capture of dysregulated or damaged cells and even cancer cells^8^. According to the “prophylaxis hypothesis”, allergic reaction is considered to be the body's way of expelling carcinogens before malignant transformation^30^. The scientific background of evolutionary biology further indicates that allergy and cancer are inversely correlated. Due to the increased awareness of hygiene, the lack of exposure to infectious agents in the industrialized countries increases sensitivity to other non-infectious agents and it brings Th2 type immune reaction against environmental factors with IgE production. That is, allergic responses could have evolved as an immunological adaptation and allergens could be substitutes for other presumed noxious agents^31^. Evolutionary biology implies that during allergic responses, released IgE engages in tumor surveillance and is exploited for tumor control in either active or passive ways^32^. Notably, the inverse relationship between cancer risk and allergic diseases appears more prominent in malignancies of tissues or organs interfacing the external environment such as cancers of the gastrointestinal tract^30^.

With regards to HCC which is categorized as a malignancy of the gastrointestinal tract, reduced risk in allergic patients can be further explained through immunological perspectives. Liver is an immune organ which contains both innate and adaptive immune cells, and plays an important role in producing cytokines and maintaining the balance between immunotolerance and activation of the immune system^33,34^. Allergic diseases induce immune responses which provoke immune effector cells to be activated and produce cytokines which leads to immunosurveillance. Previous studies suggested that stimulated immune responses by allergic diseases activate mast cells and NK cells so that it results in the protection against liver cancer^35–38^. Also, some studies indicated that released cytokines during allergic responses such as IL-6 and IL-33 could be predictive factors for survival in HCC patients^39–42^.

There are several limitations to be considered in our study. First, exposure and outcome variables were defined by using ICD-10 codes from claims data and are thus not verified by medical records. In cases of the allergic diseases, we did not analyze disease severity based on doses of drugs and laboratory data such as serum IgE levels and skin prick test. Future studies utilizing a better-defined cohort of allergic patients who are diagnosed using gold-standard methods will be needed. Since we adopted the operational definition for liver cancer from previous studies that also used NHIS database^14,15^, the definition of liver cancer could not be validated by registered medical records such as histological information, imaging or treatment. Thus, it can cause discrepancy. Future studies with more optimized definition of liver cancer are needed. Second, this study was conducted only with the South Korean population which may limit its generalizability. Future studies with a more diverse population are required to further certify the findings from this study. Third, since we could not exclude previously confirmed cases, allergic patients may have had their first diagnosis well before the index date. Therefore, there could be a bias due to the pre-existing allergic diseases. Lastly, since there may be differential distribution of sociodemographic and clinical characteristics between allergic and non-allergic groups, a propensity score matching analysis may better define the association of allergic diseases with risk of liver cancer, which awaits future studies to confirm.

Nonetheless, our study has several strengths compared to previous studies. The first study conducted by La Vecchina et al.^9^, found the protective association of history of drug allergies with primary liver cancer (OR, 0.5; 95% CI 0.20–0.90) but was only limited to acute allergies. D’Arcy et al.^10^ identified the inverse association of allergic rhinitis (aOR, 0.83; 95% CI 0.78–0.88) and asthma (aOR, 0.82; 95% CI 0.75–0.91) with liver cancer. Hemminki et al.^11^ also showed clearly decreased risk for liver cancer (SIR, 0.62; 95% CI 0.40–0.92). Although both studies covered a large population at the national level, there were no considerations for pronounced high risk factors such as HBV/HCV infection, liver cirrhosis, and alcohol consumption. In regards to smoking, both evaluated the risk of liver cancer incidence by adjusting diagnosis of Chronic Obstructive Pulmonary Disease (COPD) as a proxy for heavy smoking which could not reflect individual smoking status specifically. However, we adjusted all possible risk factors for liver cancer as follows: well-known high-risk factors such as HBV/HCV infection and history of liver cirrhosis, the number of outpatient visits during observation period, and health-related variables including body mass index, fasting serum glucose, smoking status, and alcohol consumption. HBV is the primary etiology of HCC development and HCV accounts for approximately 10% cases of HCC in South Korea^1^. Liver cirrhosis, often caused whether by alcohol abuse or by HBV infection, is also categorized as having a high risk of developing liver cancer^43,44^. Besides, alcohol-related, smoking-related and metabolic-related factors are also well associated with a greater risk of HCC in South korea^45^. Therefore, by adjusting significant high risk factors as we could, this study could represent more validated relationship between allergic disease and liver cancer compared to previous studies. Also, the length of our study duration, large size of study population, and advantage of the NHIS database further validate the reliability of our results. Since the NHIS data cover nearly all citizens in Korea, without exceptions, no participants were lost during follow-up period. The diagnostic accuracy of the Korean NHIS database for liver cancer by using ICD-10 code is quite assured especially in large-cohort studies^15^. Moreover, we further performed several sub-analyses to highlight the correlation of allergic diseases with risk of liver cancer, including sensitivity analyses.

Immunotherapy harnesses the immune system to recognize and attack cancer cells and emerging as a promising treatment for liver cancer. However, many immunotherapeutic approaches are still underway to verify its clinical applicability for the treatment of liver cancer^46^. Our findings which demonstrated the association between IgE-mediated allergic responses and decreased risk of liver cancer support the validity of immunotherapy in liver cancer and encourage more clinical trials to achieve successful treatment of liver cancer.

## Conclusions

We have elucidated the association of allergic diseases and HCC risk in detailed and specific manner by adjusting potential risk factors and conducting sub-analyses. This finding supports the rationale for utilizing immunotherapy as a potent treatment option for HCC Further investigation of allergic diseases and their role in immunosurveillance and prophylaxis against HCC may lead to development of additional therapeutic and preventative options.

## Supplementary Information

Supplementary Information.

## Data Availability

The data that support the findings of this study are available from the Korean NHIS but restrictions apply to the availability of these data, which were used under license for the current study, and so are not publicly available. Data are however available from the authors upon reasonable request and with permission of the Korean NHIS.

## Abbreviations

N: Number of patients
aHR: Adjusted hazard ratio
CI: Confidential interval
HCC: Hepatocellular carcinoma
ICC: Intrahepatic cholangiocarcinoma
SD: Standard deviation
HBV: Hepatitis B virus
HCV: Hepatitis C virus
OR: Odds ratio
aOR: Adjusted odds ratio
SIR: Standardized incidence ratio
