# Supplementary material for: Association of the presence of allergic disease with subsequent risk of liver cancer in a nationwide retrospective cohort among Koreans
Source: Sci Rep. 2022 Jun 14;12:9856. doi: 10.1038/s41598-022-14147-4 (PMC9198066; doi:10.1038/s41598-022-14147-4)

**Association of the presence of allergic disease with subsequent risk of liver cancer in a nationwide retrospective cohort among Koreans**

Ji Ah Kim^1^, Sun Jae Park^2^, Seulggie Choi^2^, Jooyoung Chang^2^, Seogsong Jeong^3^, Joseph C.Ahn^4^, Gyeongsil Lee^5^, Joung Sik Son^6,*^, Sang Min Park^2,5,*^

^1^Department of Public Health, Graduate School of Public Health, Seoul National University, Seoul, South Korea

^2^Department of Biomedical Sciences, Seoul National University Graduate School, Seoul, South Korea

^3^Department of Biomedical Informatics, CHA University School of Medicine, CHA University, Seongnam 13488, Korea

^4^Division of Gastroenterology and Hepatology, Mayo Clinic, Rochester, Minnesota

^5^Department of Family Medicine, Seoul National University Hospital, Seoul, South Korea

^6^Department of Internal Medicine, Hallym University Sacred Heart Hospital, Anyang, South Korea

**Supplementary information**

**Supplementary Table 1.** Sensitivity analysis on the association of the history of allergic diseases with risk of liver cancer incidence.

**Supplementary Table 2.** Association of allergic diseases with risk of either HCC or ICC.

**Supplementary Table 3.** Association of allergic disease incidence with risk of either HCC or ICC incidence.

**Supplementary Figure 1.** Stratified analysis on the association of the history of allergic diseases with the risk of HCC

**Supplementary Figure 2.** Stratified analysis on the association of the history of allergic diseases with the risk of ICC

**Supplementary Figure 3.** The Kaplan-Meier curve of liver cancer between allergic and non-allergic patients

**Supplementary Table 1.** Sensitivity analysis on the association of the history of allergic diseases with risk of liver cancer incidence.

|  | Non-allergic patients | Allergic patients |
| --- | --- | --- |
| Overall |  |  |
| Events, N (%) | 2,045 (0.60) | 310 (0.46) |
| Person-years | 2,890,854 | 571,420 |
| aHR (95% CI)^a^ | 1.00 (Reference) | 0.73 (0.62-0.86) |
| aHR (95% CI)^b^ | 1.00 (Reference) | 0.78 (0.69-0.88) |
| HCC |  |  |
| Events, N (%) | 1,428 (0.42) | 196 (0.29) |
| aHR (95% CI)^a^ | 1.00 (Reference) | 0.68 (0.56-0.83) |
| aHR (95% CI)^b^ | 1.00 (Reference) | 0.73 (0.63-0.86) |
| ICC |  |  |
| Events, N (%) | 402 (0.12) | 78 (0.12) |
| aHR (95% CI)^a^ | 1.00 (Reference) | 0.87 (0.63-1.19) |
| aHR (95% CI)^b^ | 1.00 (Reference) | 0.95 (0.74-1.23) |

Hazard ratio calculated by Cox proportional hazards regression analysis after adjustments for age, sex, body mass index, Charlson comorbidity index, household income, smoking, alcohol consumption, physical activity, systolic blood pressure, fasting serum glucose, total cholesterol, history of hepatitis B virus/hepatitis C virus infection, liver cirrhosis, and the number of outpatient visits

^a^ Model 1 was further adjusted by the number of outpatients visits for allergic diseases

^b^ Model 2: was further adjusted by the duration of hospitalization for allergic diseases

Acronyms: N, number of people; aHR, adjusted hazard ratio; CI, confidential interval; HCC, Hepatocellular carcinoma; ICC, intrahepatic cholangiocarcinoma.

**Supplementary** **Table 2.** Association of allergic diseases with risk of either HCC or ICC.

|  | Non-atopic dermatitis | Atopic dermatitis | Non-asthma | Asthma | Non-allergic rhinitis | Allergic rhinitis |
| --- | --- | --- | --- | --- | --- | --- |
| HCC |  |  |  |  |  |  |
| Events, N (%) | 1,615 (0.40) | 9 (0.29) | 1,554 (0.40) | 70 (0.33) | 1,493 (0.42) | 131 (0.26) |
| aHR (95% CI) | 1.00 (Reference) | 0.80 (0.42-1.55) | 1.00 (Reference) | 0.71 (0.55-0.91) | 1.00 (Reference) | 0.73 (0.60-0.87) |
| ICC |  |  |  |  |  |  |
| Events, N (%) | 475 (0.12) | 5 (0.16) | 443 (0.12) | 37 (0.17) | 430 (0.12) | 50 (0.10) |
| aHR (95% CI) | 1.00 (Reference) | 1.39 (0.57-3.36) | 1.00 (Reference) | 1.07 (0.76-1.53) | 1.00 (Reference) | 0.93 (0.69-1.26) |

Hazard ratio calculated by Cox proportional hazards regression analysis after adjustments for age, sex, body mass index, Charlson comorbidity index, household income, smoking, alcohol consumption, physical activity, systolic blood pressure, fasting serum glucose, total cholesterol, history of hepatitis B virus/hepatitis C virus infection and liver cirrhosis and the number of outpatient visits.

**Supplementary Table 3.** Association of allergic disease incidence with risk of either HCC or ICC incidence.

|  | No allergic history | Atopic dermatitis only | Asthma only | Allergic rhinitis only | Two or more allergies * |
| --- | --- | --- | --- | --- | --- |
| HCC |  |  |  |  |  |
| Events, N (%) | 1,428 (0.42) | 8 (0.35) | 57 (0.38) | 117 (0.27) | 14 (0.20) |
| aHR (95% CI) | 1.00 (Reference) | 0.86 (0.43-1.73) | 0.74 (0.56-0.98) | 0.74 (0.61-0.90) | 0.52 (0.31-0.89) |
| ICC |  |  |  |  |  |
| Events, N (%) | 402 (0.12) | 4 (0.18) | 24 (0.16) | 37 (0.09) | 13 (0.19) |
| aHR (95% CI) | 1.00 (Reference) | 1.49 (0.55-3.99) | 0.94 (0.20-1.43) | 0.84 (0.60-1.19) | 1.31 (0.73-2.36) |

Hazard ratio calculated by Cox proportional hazards regression analysis after adjustments for age, sex, body mass index, Charlson comorbidity index, household income, smoking, alcohol consumption, physical activity, systolic blood pressure, fasting serum glucose, total cholesterol, history of hepatitis B virus/hepatitis C virus infection, liver cirrhosis and the number of outpatient visits.

* Having the history of allergic diseases of either any two kinds of allergies among allergic rhinitis, atopic dermatitis, asthma or all of three allergies.

**Supplementary Figure 1.** Stratified analysis on the association of the history of allergic diseases with the risk of HCC

Hazard ratio calculated by Cox proportional hazards regression analysis after adjustments for age, sex, body mass index, Charlson comorbidity index, household income, smoking, alcohol consumption, physical activity, systolic blood pressure, fasting serum glucose, total cholesterol, history of hepatitis B virus/hepatitis C virus infection and liver cirrhosis and the number of outpatient visits.

**Supplementary Figure 2.** Stratified analysis on the association of the history of allergic diseases with the risk of ICC

Hazard ratio calculated by Cox proportional hazards regression analysis after adjustments for age, sex, body mass index, Charlson comorbidity index, household income, smoking, alcohol consumption, physical activity, systolic blood pressure, fasting serum glucose, total cholesterol, history of hepatitis B virus/hepatitis C virus infection and liver cirrhosis and the number of outpatient visits.

**Supplementary Figure 3.** Stratified analysis on the association of the history of allergic diseases with the risk of ICC


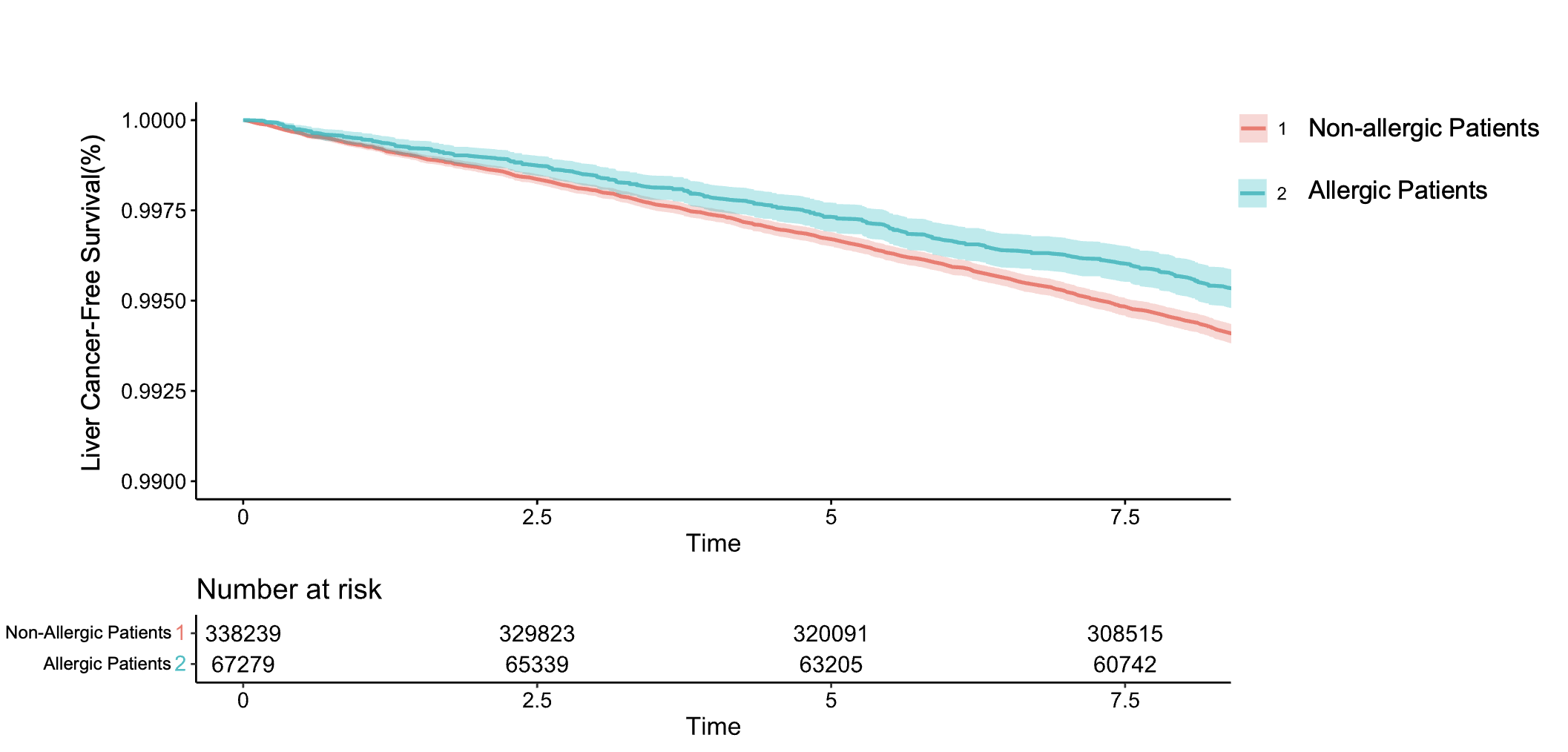

Supplement: Supplementary file 1 — Supplementary Information. [file 41598_2022_14147_MOESM1_ESM.docx]
